# Clinical Impact of a Pharmacist-Driven Prospective Audit with Intervention and Feedback on the Treatment of Patients with Bloodstream Infection

**DOI:** 10.3390/antibiotics11091144

**Published:** 2022-08-24

**Authors:** Naoto Okada, Momoyo Azuma, Kaito Tsujinaka, Akane Abe, Mari Takahashi, Yumiko Yano, Masami Sato, Takahiro Shibata, Mitsuhiro Goda, Keisuke Ishizawa

**Affiliations:** 1 Department of Pharmacy, Tokushima University Hospital, Tokushima 770-8503, Japan; 2 Department of Infection Control and Prevention, Tokushima University Hospital, Tokushima 770-8503, Japan; 3 Department of Clinical Pharmacology and Therapeutics, Tokushima University Graduate School of Biomedical Sciences, Tokushima 770-8503, Japan; 4 Clinical Research Center for Developmental Therapeutics, Tokushima University Hospital, Tokushima 770-8503, Japan

**Keywords:** antimicrobial stewardship, pharmacist, prospective audit with intervention and feedback, bloodstream infection

## Abstract

Evidence for the utility of pharmacist-driven antimicrobial stewardship programs remains limited. This study aimed to evaluate the usefulness of our institutional pharmacist-driven prospective audit with intervention and feedback (PAF) on the treatment of patients with bloodstream infections (BSIs). The effect of pharmacist-driven PAF was estimated using an interrupted time series analysis with a quasi-experimental design. The proportion of de-escalation during BSI treatment increased by 44% after the implementation of pharmacist-driven PAF (95% CI: 30–58, *p* < 0.01). The number of days of therapy decreased by 16 per 100 patient days for carbapenem (95% CI: −28 to −3.5, *p* = 0.012) and by 15 per 100 patient days for tazobactam/piperacillin (95% CI: −26 to −4.9, *p* < 0.01). Moreover, the proportion of inappropriate treatment in empirical and definitive therapy was significantly reduced after the implementation of pharmacist-driven PAF. Although 30-day mortality did not change, compliance with evidenced-based bundles in the BSI of *Staphylococcus aureus* significantly increased (*p* < 0.01). In conclusion, our pharmacist-driven PAF increased the proportion of de-escalation and decreased the use of broad-spectrum antibiotics, as well as the proportion of inappropriate treatment in patients with BSI. This indicates that pharmacist-driven PAF is useful in improving the quality of antimicrobial treatment and reducing broad-spectrum antimicrobial use in the management of patients with BSI.

## 1. Introduction

Antimicrobial resistance (AMR) is a serious concern worldwide [1]. The main cause of AMR emergence is increased selective pressure due to the inappropriate use of antimicrobial agents [2]. De-escalation strategies that minimize the use of broad-spectrum antimicrobial agents without worsening the therapeutic prognosis are important to prevent unnecessary increased selection pressure for antimicrobials [3]. Thus, the Infectious Diseases Society of America has encouraged the implementation of antimicrobial stewardship programs (ASPs), including the facilitation of de-escalation strategies [4].

Pharmacists play an essential role in the practice of ASPs. As members of the ASP team with the most medication expertise, pharmacists play a crucial role in ASPs by optimizing prescription practices, monitoring antibiotic usage, implementing infection control measures, and providing education [5]. Thus, pharmacists fill a gap in patient care and lessen the burden of the delayed treatment of optimal antibiotic therapy. A previous study reported that the treatment quality of infectious diseases was improved through pharmacist-driven ASPs with the optimization of antimicrobial agents, leading to better patient outcomes [6]. However, evidence for the utility of pharmacist-driven ASPs remains limited.

Bloodstream infection (BSI) has a poor prognosis; however, the target organisms are well defined, and treatment optimization through de-escalation or specific drug selection can be logically promoted [7]. Therefore, treatment optimization with pharmacist-driven ASPs may be particularly effective in patients with BSI. Although there have been several reports on the usefulness of pharmacist-driven ASPs in patients with BSI targeting only specific bacterial species [8,9], few reports have evaluated the utility of pharmacist-driven ASPs in patients with BSI for all bacterial species. To further clarify the effectiveness of pharmacist-driven ASPs, their effect on BSI should be assessed urgently.

Our institution established a pharmacist-driven prospective audit with intervention and feedback (PAF) for patients with BSI in August 2019 as part of an ASP. The purpose of this study is to evaluate the usefulness of pharmacist-driven PAF in patients of our practice.

## 2. Materials and Methods

### 2.1. Preintervention

Until July 2019, telephone consultations were provided by the attending physician to an infectious disease (ID) physician to support antimicrobial selection in patients with BSI. When the blood culture was positive, the results were reported by the microbiology technologist to the attending physician, who then selected the antimicrobial agents. The attending physician consulted the ID physician by telephone, only when advice on antibiotic selection was required. The ID physician provided no active advice to the attending physician. The optimization of antibiotics was left to the attending physician when the drug susceptibility of the detected bacteria was determined. The follow-up was performed only by the attending physician. If anti-methicillin-resistant *Staphylococcus aureus* (*S. aureus*) agents or carbapenems were used in the treatment, the ID physician and pharmacist reviewed the treatment weekly and discussed the choice of antibacterial agents with the attending physician when necessary. Rapid identification by matrix-assisted laser desorption/ionization time-of-flight mass spectrometry was continued from the beginning of the preintervention period.

### 2.2. Pharmacist-Driven Prospective Audit with Intervention and Feedback for BSI Patients

Pharmacist-driven PAF for patients with BSI started in August 2019. The core members of this program involved two ID physicians, one ID pharmacist who was a board-certified infection control pharmacy specialist, two certified infection control nurses, and one microbiology technologist. The ID pharmacist identified patients with positive blood cultures from electronic medical records on each weekday. Culture results were also reported by the attending physician by a microbiology technologist. The ID pharmacist audited the treatment for BSI each weekday. The ID pharmacist reviewed the following problems: (1) inappropriate selection, dosing, or duration of antibiotics; (2) lack of appropriate de-escalation; and (3) failure to collect bacterial cultures. On the day after the pharmacist identified the problem cases, the ID pharmacist discussed with the attending physician about the selection of antibiotics or de-escalation based on the pathogen, optimal dose, possibility of contamination, or addition of culture testing to optimize the treatment.

Pharmacist-driven PAF was continued until infection remission or patient discharge from the hospital with or without ID pharmacist intervention. The ID pharmacist consulted the ID physicians regarding antimicrobial treatment of BSI patients during the implementation of PAF. Telephone consultations provided by attending physicians were also accepted. Team conferences with program members were held once a week to review audited patients, and treatment details were confirmed by program members. In addition to the PAF, the ID pharmacist and physicians provided lectures on the proper use of antimicrobials to all physicians on a regular basis (twice a year).

### 2.3. Study Population

This single-center study was conducted in our hospital using data from August 2017 to July 2021. Patients whose blood cultures were positive between August 2017 and July 2019 and between August 2019 and July 2021 were classified into the preintervention and intervention groups, respectively. Patients were excluded if the detected bacteria were contaminated, which were defined as (1) patients in whom *Bacillus* spp., *Corynebacterium* spp., *Propionibacterium* spp., *Micrococcus* spp., or coagulase-negative *staphylococci* were isolated from only one blood culture or (2) patients in whom the patient’s condition recovered without the administration of antibiotics [10]. Patients aged < 18 years who died within 72 h after the blood culture was positive or who were not hospitalized were also excluded. Only the patients’ first episode of positive blood culture during the study period was included in the analysis. The following baseline information was extracted from the electronic medical records: age, sex, serum creatinine level, estimated glomerular filtration rate (eGFR, per 1.73 m^2^), history of diabetes mellitus, primary disease treatment department, bacteria identified in blood culture, history of antimicrobial therapy for BSI treatment, and Pitt bacteremia score at the time of positive blood culture. The estimation formula was used to calculate eGFR, and an eGFR less than 30 mL/min/1.73 m^2^ was defined as stage 4 chronic kidney disease [11]. The Pitt bacteremia score was calculated based on temperature, blood pressure, respiration, cardiac arrest, and mental condition, and a score of 4 or more was considered to indicate severe bacteremia [12].

### 2.4. Outcome Measures

The primary outcome measures were the (1) proportion of de-escalation and inappropriate treatment in empirical and definitive therapy and (2) days of therapy (DOT) per 100 patient days of carbapenem and tazobactam/piperacillin. The secondary outcome measures were all-cause 30-day mortality in all patients and in patients with BSI with *S. aureus* or *Candida* spp. The compliance of the evidence-based bundle was also evaluated in patients with BSI with *S. aureus* or *Candida* spp.

Antibiotic de-escalation therapy was defined as the discontinuation of at least one antibiotic, the replacement of empirical broad-spectrum antibiotics with narrow-spectrum antibiotics, or switch to oral administration based on blood culture results [13]. The proportion of de-escalation was defined as the percentage of patients with BSI who underwent de-escalation until the end of BSI treatment. Nonrecommended empiric therapy was defined as the administration of an antibiotic that was naturally resistant to the detected organism until drug susceptibility was determined. Nonrecommended definitive therapy treatment was defined as the continued administration of resistant antimicrobial agents or a shorter duration of antimicrobial therapy than recommended. The recommended duration of antimicrobial therapy was defined as at least 2 weeks for *S. aureus* and *Candida* spp. and at least 1 week for other species [14,15,16]. The DOT per 100 patient days of carbapenem or tazobactam/piperacillin was calculated by multiplying the total days of each antimicrobial administration for BSI treatment divided by the total number of BSI treatment days by 100. All-cause 30-day mortality was defined as the percentage of patients with BSI who died within 30 days of a positive blood culture. An evidence-based bundle for BSI of *S. aureus* was defined according to four items: re-test of blood culture, conduct of echocardiography, source control, and definitive therapy with an optimal antimicrobial agent [8]. An evidence-based bundle for BSI of *Candida* spp. was defined according to four items: re-test of blood culture, consultation with ophthalmology, source control, and definitive therapy with an optimal antifungal agent [17].

### 2.5. Statistical Analyses

Differences between the two groups were analyzed using the parametric unpaired *t*-test or nonparametric Mann–Whitney U test, as applicable. Data distribution was evaluated using the Shapiro–Wilk test. The parametric test was used in the case of a normal distribution, and the nonparametric test was used in the case of a non-normal distribution. Chi-square or Fisher’s exact tests were used to analyze the nominal scales. An interrupted time series analysis (ITS) was performed to identify the effect of pharmacist-driven PAF on the rate of de-escalation and DOT of carbapenem and tazobactam/piperacillin. Twenty-four monthly data points in both pre- and post-intervention periods and a total of forty-eight monthly data points were used for ITS analysis. Change level and slope were modeled using the following segmented regression model: the dependent variable was the outcome and independent variables were the indicators representing before and after intervention, the time elapsed since the start of the study, and the time elapsed since the intervention. No transition period or comparison group was established. In addition, regression models for sensitivity analysis were constructed by adding a harmonic term to account for seasonality. All statistical analyses were performed using the R statistical software version 4.0.2 (https://www.r-project.org/). For ITS analysis, the tsModel package was used. In all analyses, a two-tailed *p* value of <0.05 was considered significant.

## 3. Results

### 3.1. Patient Characteristics

**Figure 1:**
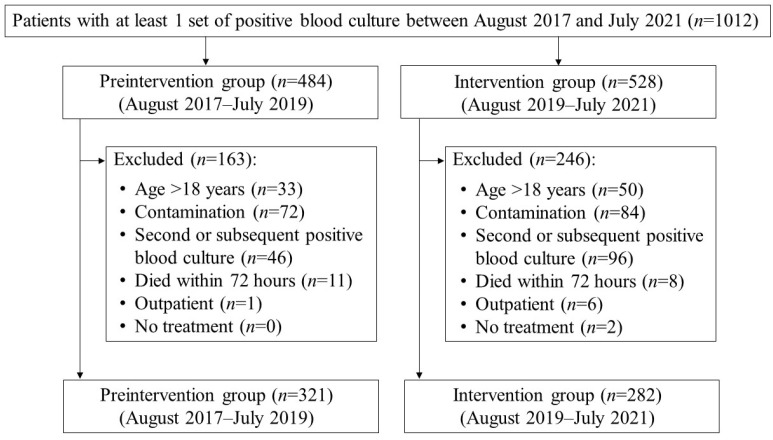
Flowchart of patient selection. Pharmacist-driven PAF was initiated in August 2019. The patients after this date are classified into the intervention group.

Of the 1012 patients whose blood cultures were positive during the study period, 603 patients were included in the analysis. Among them, 321 and 282 patients were classified into the preintervention and intervention groups, respectively (Figure 1). Age, sex, eGFR, history of diabetes mellitus, primary disease treatment department, bacteria identified in blood culture, and Pitt bacteremia score were not significantly different between the two groups (Table 1). The number of patients with a Pitt bacteremia score of ≥4 also did not differ between the two groups.

### 3.2. Primary Outcome Measures

The primary outcome measures are presented in Table 2. The proportion of patients who used tazobactam/piperacillin as empiric therapy was significantly higher in the intervention group (*p* = 0.01). The proportion of de-escalation implementation was also significantly higher in the intervention group (*p* < 0.01). The type of de-escalation did not differ between the two groups. The DOT of carbapenem and tazobactam/piperacillin were significantly lower in the intervention group (*p* < 0.01). Moreover, the proportion of nonrecommended empiric and definitive therapy was significantly lower in the intervention group (*p* < 0.01).

**Figure 2:**
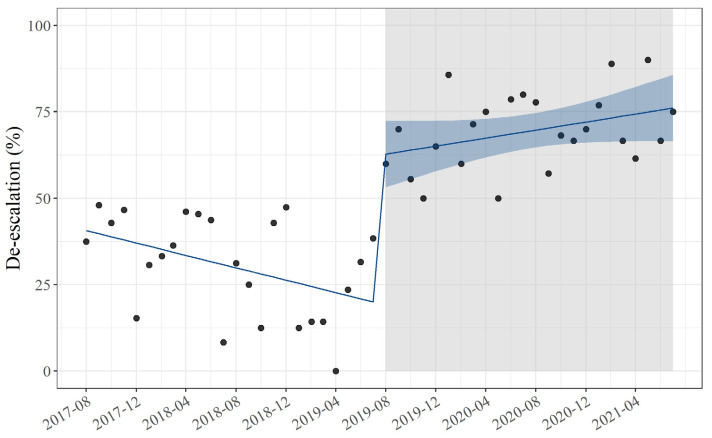
ITS analysis for the proportion of de-escalation. The gray area indicates the start of pharmacist-driven PAF. The dots indicate the measured values for each month, and the blue line indicates the regression line. The light blue band indicates 95% confidence interval. Abbreviation: ITS, interrupted time series; PAF, prospective audit with intervention and feedback.

**Figure 3:**
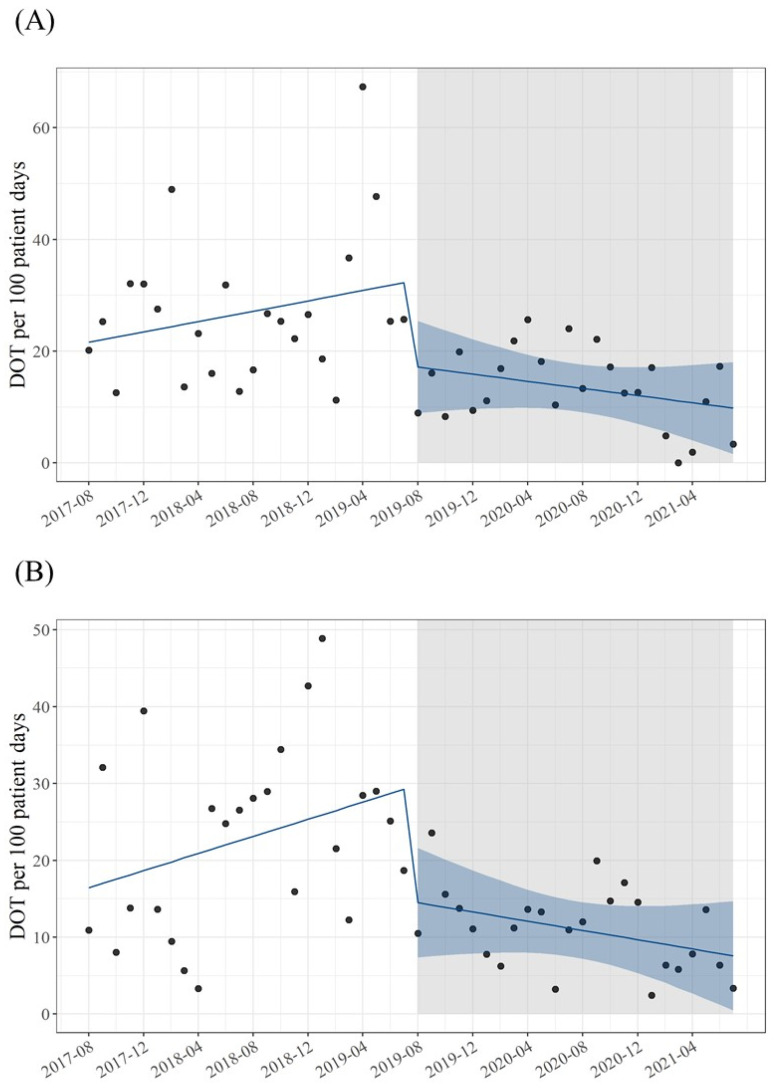
ITS analysis for DOT of carbapenem or tazobactam/piperacillin. (**A**), DOT of carbapenem; (**B**), DOT of tazobactam/piperacillin. The gray area indicates the start of pharmacist-driven PAF. The dots indicate the measured values for each month, and the blue line indicates the regression line. The light blue band indicates 95% confidence interval. Abbreviation: ITS, interrupted time series; PAF, prospective audit with intervention and feedback.

ITS analysis was performed to determine the impact of pharmacist-driven PAF on de-escalation practices and the DOT of carbapenems and tazobactam/piperacillin (Table 3). The proportion of de-escalation after pharmacist-driven PAF significantly increased (level change: 44%, 95% CI: 30–58, *p* < 0.01). Compared with the pre-trend slope, the post-trend slope in the proportion of de-escalation per month was significantly lower after pharmacist-driven PAF (slope change: 1.5, 95% CI: 0.46–2.5, *p* < 0.01) (Figure 2). The DOT of carbapenem and tazobactam/piperacillin after pharmacist-driven PAF significantly decreased (level change: −16, 95% CI: −28 to −3.5, *p* = 0.12 and level change: −15, 95% CI: −26 to −4.9, *p* < 0.01, respectively). Compared with the pre-trend slopes, the post-trend slopes in DOT per month of carbapenem and tazobactam/piperacillin were significantly lower after pharmacist-driven PAF (slope change: −0.78, 95% CI: −1.6–0.08, *p* = 0.076 and slope change: −0.86, 95%CI: −1.6 to −0.11, *p* = 0.026, respectively) (Figure 3). Similar results were obtained in the sensitivity analysis using a model that accounted for seasonality (Supplemental Table S1, Supplemental Figures S1 and S2).

### 3.3. Secondary Outcome Assessment

The secondary outcomes are presented in Table 4. The proportion of all-cause 30-day mortality in all patients and in BSI patients with *S. aureus* or *Candida* spp. was not significantly different between the two groups. In BSI with *S. aureus*, the compliance of the evidence-based bundles with re-blood culture, source control, and optimal antimicrobial agents was significantly higher in the intervention group (*p* < 0.01). Meanwhile, there were no significant between-group differences in the compliance of the evidence-based bundle in BSI with *Candida* spp.

## 4. Discussion

This study showed that pharmacist-driven PAF promoted a de-escalation strategy, reduced inappropriate broad-spectrum antimicrobial administration, and increased compliance with evidence-based bundles in patients with BSI. Moreover, pharmacist-driven PAF reduced the use of carbapenems and tazobactam/piperacillin. These results indicate that pharmacist-driven PAF can improve treatment quality by optimizing antimicrobial therapy in patients with BSI.

The important role of pharmacists in optimizing the selection and dosage of antimicrobial agents in ASPs has been emphasized [5]. Several meta-analyses have reported that pharmacist-driven ASPs can lead to a more appropriate prescription of antimicrobial agents [18,19,20,21]. However, the utility of pharmacist-driven ASPs in patients with BSI remains limited. Shinoda et al. reported that pharmacist-led ASPs in patients with injectable antimicrobial treatment led to the optimization of antimicrobial treatment in BSI with *E. coli* [9]. Kufel et al. also reported that pharmacist-driven ASPs for BSI patients with *S. aureus* infection improved compliance with evidence-based bundles [8]. We conducted a pharmacist-driven PAF for BSI patients with all bacteria as part of the ASPs and found that this program promoted antimicrobial optimization. This evidence suggests that the quality of BSI treatment can be improved by pharmacist-driven PAF, regardless of the bacterial species. It has been noted that the human resources of ID physicians are limited, and the implementation of ASPs by ID physicians universally is difficult [22]. Meanwhile, pharmacists, who have more human resources devoted to ASPs [23], are expected to be leaders in promoting ASPs. ASPs should be implemented universally and promoting pharmacist-driven PAF is reasonable with respect to human resources. Our results support the usefulness of pharmacist-driven PAF.

The optimization of treatment quality by our program was supported by the promotion of de-escalation and an increase in the recommended empiric and definitive therapy. Given that an inappropriate increase in selective pressure with broad-spectrum antimicrobial agents is an influencing factor of AMR [2], the promotion of de-escalation is beneficial for AMR control. In this study, there was no significant difference in the type of de-escalation between the intervention and nonintervention groups. Although switching to oral antibiotics may be beneficial in reducing medical costs [24], the proportion of patients with an oral switch was not different between the two groups. Furthermore, “shorter is better” is now a common suggestion in relation to antimicrobial use [25], but shortening the duration of antimicrobial therapy was not observed in the current study (14.5 days in the preintervention group and 13.9 days in the intervention group). Inappropriate oral switching or shorter treatment duration may increase the risk of relapse, and thus these strategies should be applied cautiously. It is assumed that the number of oral switches or shorter treatment cases did not increase because of unclear criteria for these strategies. If evidence on oral switching and shortening the treatment period accumulates, further effects of pharmacist-driven PAF are expected.

Our pharmacist-driven PAF decreased the use of carbapenems and tazobactam/piperacillin in patients with BSI. As a recommendation of carbapenem-sparing regimens increases other broad-spectrum antibiotics usage [26], reducing multiple broad-spectrum antibiotics usage is important to prevent unnecessary increased selection pressure for antimicrobials. In particular, tazobactam/piperacillin is a broad-spectrum antibacterial agent routinely used in the treatment of infectious diseases, and the promotion of carbapenem-sparing regimens increases tazobactam/piperacillin use [27]. Therefore, it is important to monitor the use of tazobactam/piperacillin in addition to carbapenems when performing ASPs. Significantly more patients in the intervention group used tazobactam/piperacillin empirically, but significantly fewer DOT of tazobactam/piperacillin was observed. This reflects the promotion of de-escalation strategies. The ITS analysis also showed that the pharmacist-driven PAF not only had an immediate effect on reducing broad-spectrum antimicrobial usage, but also induced a long-term effect of changing usage trends. Although the effect of a decrease in broad-spectrum antimicrobial use is often observed in the implementation of ASPs [27], it is noteworthy that pharmacist-driven PAFs have produced sustained changes in usage trends. Pharmacist-driven PAF may be a useful strategy for a consistent decrease in broad-spectrum antibiotics, leading to AMR control.

There was no difference in all-cause 30-day mortality between the two groups. As the all-cause 30-day mortality in the preintervention group was 6.5%, lower than that in a previous report [28], our pharmacist-driven PAF could not reduce mortality compared with that in the preintervention group. Compliance with evidence-based bundles in BSI with *S. aureus* increased after the implementation of pharmacist-driven PAF. Previous studies have also reported an increase in bundle compliance rates with ASP implementation [8]. Our results support the possibility that pharmacist-driven PAF can improve the quality of treatment of BSI caused by *S. aureus*. Meanwhile, we could not detect a difference in the compliance rate of the evidence-based bundle for candidemia patients between the two groups, possibly because of the small sample size. Further studies are needed to evaluate the effects of pharmacist-driven PAF on candidemia treatment.

This study had some limitations. First, this is a single-center retrospective study. Given that antimicrobial selection is influenced by institutional policies, a prospective multicenter study is needed to generalize the utility of pharmacist-driven PAF. Second, it is possible that the characteristics of the analyzed population changed over time. The Japanese Ministry of Health, Labor, and Welfare published a manual for antimicrobial stewardship in 2017 [29]. We cannot deny that physician attitudes toward infectious disease treatment have changed over time, which may have led to an overestimation of the effectiveness of pharmacist-driven PAF in this study. However, because our program also educated all physicians on the proper use of antimicrobials, any change in physician practice behavior might be attributed to pharmacist-driven PAF. Moreover, the same results were obtained in the model that considered seasonality in ITS analysis. This model can control autocorrelation in the target population [30], suggesting that the accuracy of the ITS model is robust. Third, although the coronavirus disease 2019 (COVID-19) pandemic may have influenced antimicrobial selection, there were no BSIs in COVID-19 patients at our institution. Finally, the entry of bacteria was not diagnosed in some patients, and factors that determine the duration of antimicrobial therapy (e.g., the availability of drainage) could not be fully investigated. This study could serve as a reference for further research to resolve these limitations.

## 5. Conclusions

Our pharmacist-driven PAF facilitated a de-escalation strategy, reduced the use of carbapenem and tazobactam/piperacillin and increased compliance with evidence-based bundles in the treatment of patients with BSI. This evidence indicates that our program could improve the quality of antimicrobial therapy and reduce the use of broad-spectrum antimicrobial agents. With respect to AMR control and human resources, pharmacist-driven ASPs should be promoted, and the evidence presented in this study provides strong support for the utility of pharmacist-driven PAF.

## Figures and Tables

**Table 1:** Patient characteristics.

Variables	Preintervention Group (*n* = 321)	Intervention Group (*n* = 282)	*p*-Value
Age (years), mean (SD)	67.05 (14.22)	66.90 (14.29)	0.85 ^a^
Male sex (%)	199 (62.0)	174 (61.7)	1.00 ^b^
eGFR (ml/min/1.73 m^2^), mean (SD)	65.71 (35.04)	67.65 (41.58)	0.55 ^a^
Chronic kidney disease stage 4, *n* (%)	44 (13.7)	49 (17.4)	0.22 ^b^
Diabetes mellitus, *n* (%)	100 (31.2)	100 (35.5)	0.30 ^b^
Department, *n* (%)			
Gastroenterology	52 (16.2)	51 (18.1)	0.54 ^b^
Gastrointestinal surgery	40 (12.5)	35 (12.4)	0.99 ^b^
Hematology	44 (13.7)	35 (12.4)	0.64 ^b^
Respiratory	31 (9.6)	28 (10.0)	0.91 ^b^
Urology	29 (9.0)	26 (9.2)	0.94 ^b^
Neurosurgery	20 (6.2)	10 (3.5)	0.13 ^b^
Obstetrics and gynecology	11 (3.4)	18 (6.4)	0.091 ^b^
Orthopedic surgery	2 (0.6)	8 (2.8)	0.051 ^c^
Others	92 (28.7)	71 (25.2)	0.34 ^b^
Pathogen, *n* (%)			
Methicillin-sensitive *Staphylococcus aureus*	26 (8.1)	18 (6.4)	0.42 ^b^
Methicillin-resistant *Staphylococcus aureus*	9 (2.8)	7 (2.5)	0.81 ^b^
Coagulase-negative staphylococci	52 (16.2)	39 (13.8)	0.42 ^b^
*Streptococcus* spp.	29 (9.0)	16 (5.7)	0.12 ^b^
*Enterococcus* spp.	17 (5.3)	21 (7.4)	0.28 ^b^
*E. coli*	35 (10.9)	41 (14.5)	0.18 ^b^
ESBL-producing *E. coli*	14 (4.4)	21 (7.4)	0.11 ^b^
NDM-1-producing *E. coli*	1 (0.3)	0 (0)	1.00 ^c^
*Klebsiella* spp.	38 (11.8)	21 (7.4)	0.075 ^b^
*Enterobacter* spp.	15 (4.7)	15 (5.3)	0.72 ^b^
*Acinetobacter* spp.	9 (2.8)	4 (1.4)	0.27 ^c^
*Serratia* spp.	8 (2.5)	5 (1.8)	0.54 ^c^
*Pseudomonas aeruginosa*	10 (3.1)	10 (3.5)	0.77 ^b^
*Candida* spp.	8 (2.5)	7 (2.5)	0.99 ^b^
Mixed infection	26 (8.1)	23 (8.2)	1.00 ^b^
Others	24 (7.5)	34 (12.1)	0.057 ^b^
Pitt bacteremia score (IQR)	1 (0–2)	1 (0–2)	0.351 ^a^
Pitt bacteremia score ≥ 4 (%)	35 (10.9)	29 (10.3)	1.00 ^b^

Data are expressed as the mean (SD), median (IQR), or number of patients (%). ^a^: Mann–Whitney U test. ^b^: chi-square test. ^c^: Fisher’s exact test. Abbreviations: IQR, interquartile range; SD, standard deviation; eGFR, estimated glomerular filtration rate; E. coli, Escherichia coli; ESBL, extended-spectrum beta-lactamase; NDM, New Delhi metallo-beta-lactamase.

**Table 2:** Primary outcome assessment.

Treatment	Preintervention Group (*n* = 321)	Intervention Group (*n* = 282)	*p*-Value
Empiric therapy, *n* (%)			
Carbapenem	67 (20.9)	64 (22.7)	0.59 ^a^
Tazobactam/piperacillin	87 (27.1)	104 (36.9)	0.01 ^a^
De-escalation, *n* (%)	104 (32.4)	193 (68.4)	<0.01 ^a^
Switch to narrow-spectrum agents	84 (80.8)	149 (77.2)	0.48 ^a^
Switch to oral agents	12 (11.5)	21 (14.1)	0.86 ^a^
Stop	8 (7.7)	23 (11.9)	0.26 ^a^
DOT per 100 patient days, mean (SD)			
Carbapenem	26.9 (13.1)	13.5 (6.9)	<0.01 ^b^
Tazobactam/piperacillin	22.3 (12.5)	11.7 (5.3)	<0.01 ^c^
Nonrecommended empiric therapy (%)	30 (9.3)	0 (0)	<0.01 ^a^
Nonrecommended definitive therapy (%)	82 (25.5)	17 (6.0)	<0.01 ^a^

Data are expressed as the mean (SD) or number of patients (%). ^a^: chi-square test. ^b^: Mann–Whitney U test. ^c^: unpaired *t*-test. Abbreviation: DOT, days of therapy.

**Table 3:** Parameters of ITS analysis.

Parameter	Estimate	95% CI	*p*-Value
De-escalation			
Level change	44	30, 58	<0.01
Slope change	1.5	0.46, 2.5	<0.01
DOT of carbapenem			
Level change	−16	−28, −3.5	0.012
Slope change	−0.78	−1.6, 0.08	0.076
DOT of tazobactam/piperacillin			
Level change	−15	−26, −4.9	<0.01
Slope change	−0.86	−1.6, −0.11	0.026

Abbreviations: ITS, interrupted time series; DOT, days of therapy; CI, confidence interval.

**Table 4:** Secondary outcome assessment.

Outcome	Preintervention Group	Intervention Group	*p*-Value
30-day mortality	21 (6.5)	20 (7.1)	0.79 ^c^
*Staphylococcus aureus* ^a^	1 (2.8)	3 (11.1)	0.31 ^d^
*Candida* spp.^b^	2 (25.0)	0 (0)	0.15 ^d^
Bundle compliance			
*Staphylococcus aureus* ^a^			
Re-test of blood culture	18 (50.0)	25 (92.6)	<0.01 ^c^
Echocardiography	16 (44.4)	15 (55.6)	0.38 ^c^
Source control	19 (52.8)	24 (88.9)	<0.01 ^c^
Optimal antimicrobial agents	26 (72.2)	27 (100.0)	<0.01 ^c^
*Candida* spp.^b^			
Re-test of blood culture	5 (62.5)	11 (91.7)	0.26 ^c^
Consult to ophthalmology	4 (50.0)	7 (58.3)	1.00 ^d^
Source control	6 (75.0)	6 (50.0)	0.37 ^c^
Optimal antifungal agents	7 (87.5)	12 (100.0)	0.40 ^c^

Data are expressed as the number of patients (%). ^a^: Number of patients with *Staphylococcus aureus* BSI: preintervention group, *n* = 36; intervention group, *n* = 27. ^b^: Number of patients with *Candida* spp. BSI: preintervention group: *n* = 8; intervention group: *n* = 12. ^c^: chi-square test. ^d^: Fisher’s exact test.

## Data Availability

Not applicable.

## Supplementary Materials

The following supporting information can be downloaded at: https://www.mdpi.com/article/10.3390/antibiotics11091144/s1, Figure S1: Sensitivity analysis for the proportion of de-escalation; Figure S2: Sensitivity analysis for DOT of carbapenem and tazobactam/piperacillin; Table S1: Parameters of sensitivity analysis.
