# Supplementary material for: Clinical Impact of a Pharmacist-Driven Prospective Audit with Intervention and Feedback on the Treatment of Patients with Bloodstream Infection
Source: Antibiotics (Basel). 2022 Aug 24;11(9):1144. doi: 10.3390/antibiotics11091144 (PMC9495130; doi:10.3390/antibiotics11091144)
Supplement: Supplementary file 1 [file antibiotics-11-01144-s001.zip › Supplemental file1.pdf]

Table S1. Parameters of sensitivity analysis.

Parameter	Estimate	95% CI	p-value
De-escalation			
Level change	47	31, 62	<0.01
Slope change	1.5	0.44, 2.5	<0.01
DOTs of carbapenem			
Level change	-13	-27, -0.03	0.049
Slope change	-0.78	-1.7, 0.10	0.079
DOTs of tazobactam/piperacillin			
Level change	-19	-30, -8.5	<0.01
Slope change	-0.86	-1.6, -0.14	0.020
