# Supplementary material for: Clinical Impact of a Pharmacist-Driven Prospective Audit with Intervention and Feedback on the Treatment of Patients with Bloodstream Infection
Source: Antibiotics (Basel). 2022 Aug 24;11(9):1144. doi: 10.3390/antibiotics11091144 (PMC9495130; doi:10.3390/antibiotics11091144)
Supplement: Supplementary file 1 [file antibiotics-11-01144-s001.zip › Supplemental file2.pdf]

Figure S1. Sensitivity analysis for the proportion of de-escalation.

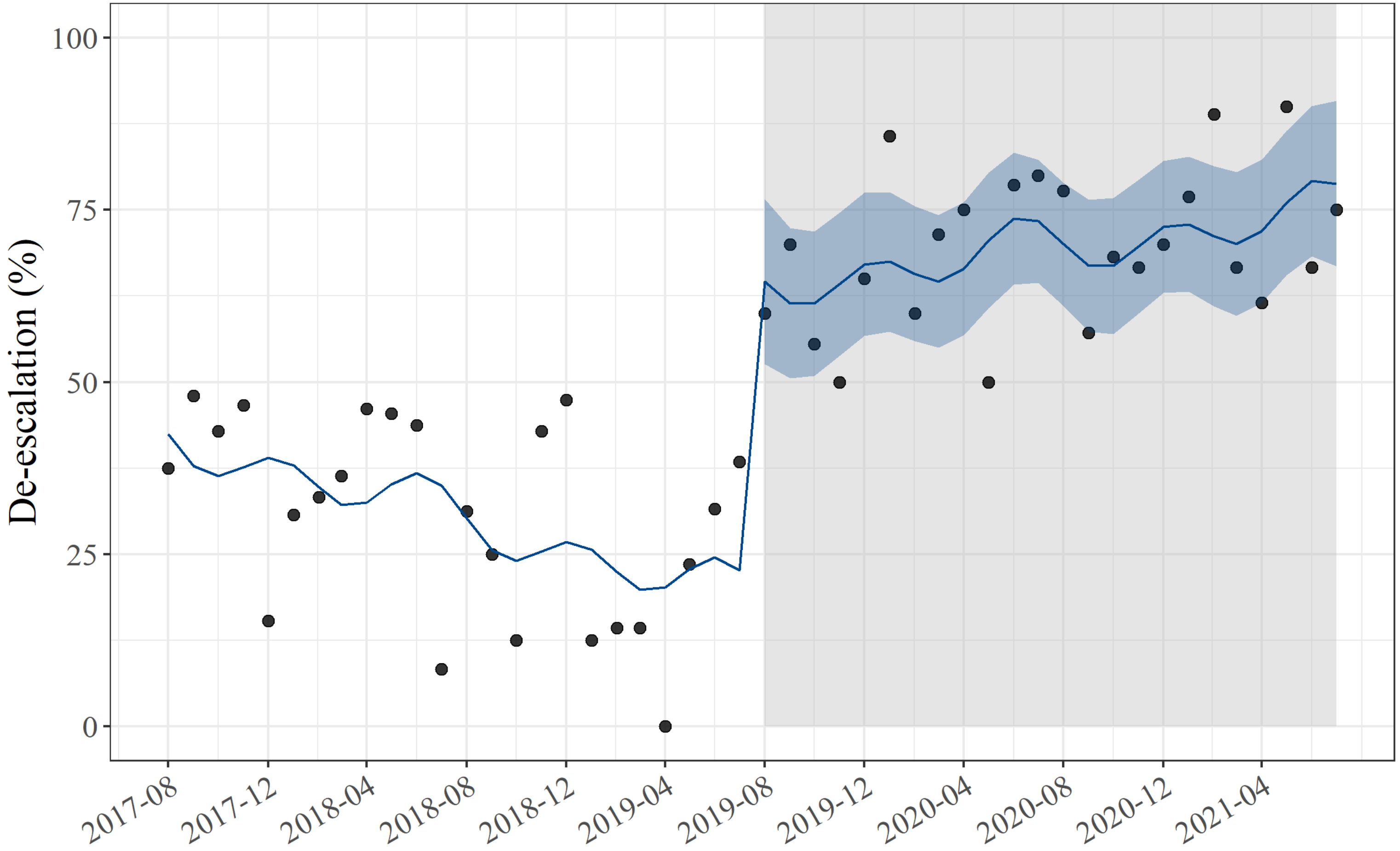

The gray area indicates the start of pharmacist-driven PAF.

The dots indicate the measured values for each month, and the blue line indicates the regression line. The light blue band indicates 95% confidence interval.

Abbreviation: PAF, prospective audit with intervention and feedback
