# Supplementary material for: Clinical Impact of a Pharmacist-Driven Prospective Audit with Intervention and Feedback on the Treatment of Patients with Bloodstream Infection
Source: Antibiotics (Basel). 2022 Aug 24;11(9):1144. doi: 10.3390/antibiotics11091144 (PMC9495130; doi:10.3390/antibiotics11091144)
Supplement: Supplementary file 1 [file antibiotics-11-01144-s001.zip › Supplemental file3.pdf]

Figure S2. Sensitivity analysis for DOT of carbapenem and tazobactam/piperacillin.

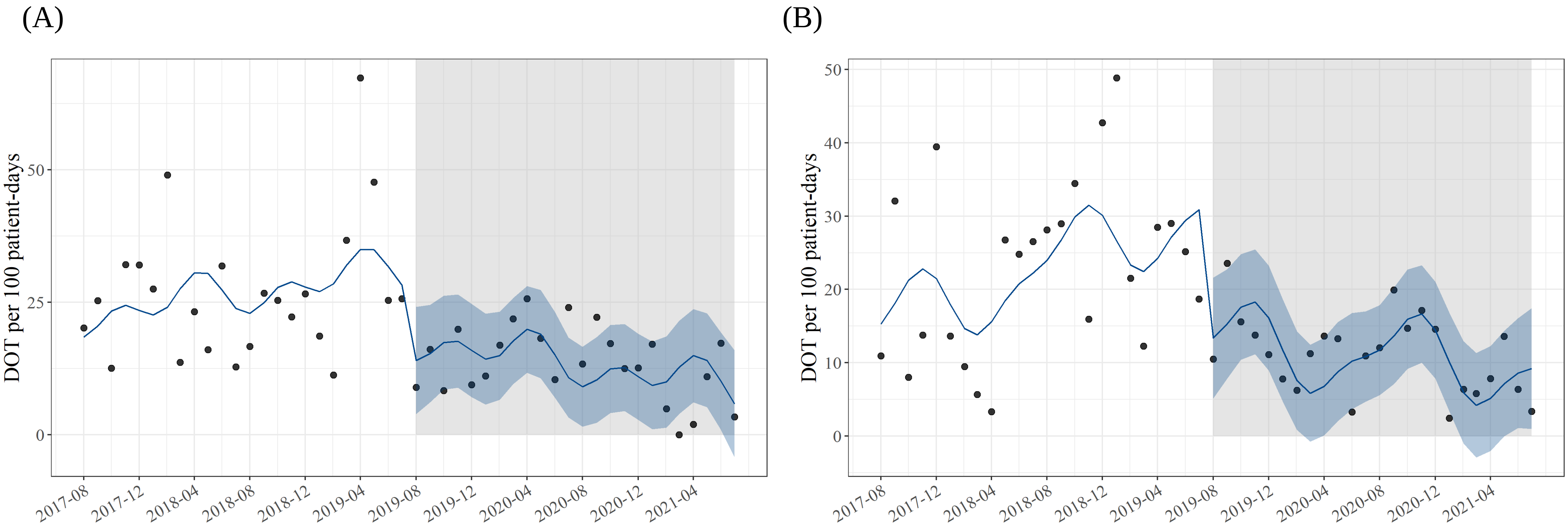

A, DOT of carbapenem; B, DOT of tazobactam/piperacillin.

The gray area indicates the start of pharmacist-driven PAF. The dots indicate the measured values for each month, and the blue line indicates the regression line.

The light blue band indicates 95% confidence interval.

Abbreviation: DOT, days of therapy; PAF, prospective audit with intervention and feedback
